# Modelling of Musical Perception using Spectral Knowledge Representation

**DOI:** 10.5334/joc.356

**Published:** 2024-04-08

**Authors:** Steven T. Homer, Nicholas Harley, Geraint A. Wiggins

**Affiliations:** 1 Computational Creativity Lab, Artificial Intelligence Research Group, Vrije Universiteit Brussel, Belgium; 2 Cognitive Science Research Group, School of Electronic Engineering and Computer Science, Queen Mary University of London, UK

**Keywords:** music perception, spectral analysis, key affinity, key distance, resonance, cognitive modelling, knowledge representation, Hilbert space, neural dynamics

## Abstract

We present a novel approach to representing perceptual and cognitive knowledge, *spectral knowledge representation*, that is focused on the oscillatory behaviour of the brain. The model is presented in the context of a larger hypothetical cognitive architecture. The model uses literal representations of waves to describe the dynamics of neural assemblies as they process perceived input. We show how the model can be applied to representations of sound, and usefully model music perception, specifically harmonic distance. We demonstrate that the model naturally captures both pitch and chord/key distance as empirically measured by Krumhansl and Kessler, thereby providing an underlying mechanism from which their toroidal model might arise. We evaluate our model with respect to those of Milne and others.

## 1 Introduction

We present the first results from a novel approach to the representation of knowledge in Artificial Intelligence (AI) systems, *spectral knowledge representation*. Our approach is motivated by results from the research on the perception and cognitive processing of music, and fits within a broader framework, the Information Dynamics of Thinking (IDyOT: Wiggins, 2020). IDyOT proposes a multilayer cognitive architecture that models at the level of cognitive function, inspired by neural implementation. It differs from many cognitive architectures in that modelling is focused on the emergent, bottom-up properties of a small set of mathematical structures when applied to data, rather than designed top-down as a system. IDyOT is based on four fundamental operations: segmentation (in time), categorisation (in percept and meaning), prediction (of anticipated percepts and meanings), and abstraction (of significant components of percept and meaning across time) (Wiggins, 2020). These operations are formalized using principles of information theory (Shannon, 1948) and differential geometry (Needham, 2021), but in order to work in concert, they must share a base representation^1^ of perceptual information. Spectral knowledge representation is that base. Here, we show how our base representation captures musical auditory information with the goal of providing a motivating example of the general method, focusing solely on the spectral representational aspect of IDyOT, instead of its operational components.

For the current article, we begin from recent understanding that the experience of hearing sound corresponds with electrophysiological analogues of that sound in the brain (Kraus and Nicol, 2019). For this to be so, some neural assemblies must be oscillating in order to produce that wave. We posit that this is an example of the brain representing sensory experience, an event which may be remembered and later recalled. We model this electrophysiological representation using mathematical structures called *resonances* (Section 3.2). For our current purpose, a key feature of IDyOT is that knowledge representation and recall in the human brain are viewed as a process of *oscillation* and *sympathetic resonance*, respectively. Thus, information is encoded in a neural assembly which oscillates with a particular waveform when active. Recall occurs when such an assembly is stimulated into oscillation by another similar^2^ oscillation.

Resonances are damped or driven complex oscillators, such as commonly arise in the context of dynamical systems. When a system can be formulated as a set of (stochastic) first-order differential equations, resonances are the set of coupled oscillators that describe its non-equilibrium steady state behaviour. For instance, the cochlea in the inner ear is often modeled as a dynamical system (e.g., Lindeberg and Friberg, 2015; Lerud et al., 2019a). The cochlea being the primary transduction site for sound from the environment into the brain, these models indicate that resonances may be a good base representation, at least for sound information. More generally, in neural dynamics, functional connectivity is governed by the neural states and topology of the effective connectivity (Friston et al., 2014). Functional connectivity is the observed dependence or covariance between brain regions, e.g., from an fMRI, whereas effective connectivity is the underlying stochastic dynamical network generating the observed fluctuations (Friston, 2011). Resonances appear here too, where the resonant frequencies correspond to the eigenvalues of the effective connectivity and the resonant amplitudes correspond to the projection of initial conditions onto the eigenfunctions. This is important since it indicates that neural dynamics in the brain are naturally represented using resonances.

Further, we show that all normalizable continuous functions may be arbitrarily well approximated by a linear combination of resonances, and for a discrete sequence of data points, the resonance representation is unique. Therefore, we propose that resonances can be a good alternative to the ubiquitous Fourier basis for representing signals in *L^2^* Hilbert space (Kennedy and Sadeghi, 2013). Though we have devised techniques to calculate resonances from data (Homer et al., forthcoming), producing what we call a *Discrete Resonance Spectrogram* (DRS), details of that analysis are beyond the scope of the current paper, so we take resonances as input to our perceptual model as if provided by an oracle.

As vectors in a Hilbert space, resonances can be related through distance and angles, analogous to the pitch and tonal spaces that are familiar to music psychologists (Longuet-Higgins, 1962a; b; Balzano, 1977; Lerdahl, 2001). Following the seminal music theory of Rameau (1722), we show how resonance representations of pitches and chords allow their perceptual similarity to be modeled using operators on the Hilbert space of resonances, mirroring other related work surveyed in Section 2.2. We suggest that such operators are candidates as cognitive models for pitch perception, and therefore compare them with other dominant models in the literature (Krumhansl and Kessler, 1982; Lerdahl, 2001; Milne et al., 2015) showing a competitive correlation with empirical studies.

In contrast with music-theoretic models, and in the same sense as that of the spectral pitch class model of Milne et al. (2015), we believe that we may reasonably claim that our model is *explanatory* (in contrast with a *descriptive* one: Wiggins, 2011), in that it proposes an underlying mechanism by which the empirical data might be calculated in the brain. Furthermore, like the spectral pitch class model, it unifies key perception (expressed as descriptive empirical tonal profiles: Krumhansl and Kessler, 1982) and chord or tonal difference (expressed as descriptive musicological tonal distance: Lerdahl, 2001).

This article is structured as follows. First, we introduce the idea of spectral knowledge representation in context of cognitive architecture and knowledge representation research in artificial intelligence in general. Next we describe resonance representations and their relationship to other orthonormal representations. Then, we show how our technique simulates pitch and chord similarity in tonal pitch space, comparing with the models mentioned above. We conclude with a summary and some targets for future work.

## 2 Background

### 2.1 Cognitive Architecture Research

Cognitive architecture research (e.g., Vernon, 2014; Baars, 1988; Wiggins, 2020) is an interdisciplinary research field that applies a systems level approach to understanding the function of cognitive entities. That is, rather than apply reductionist science to focus on specific phenomena using controlled experiments, as is usual in cognitive science, it proceeds by large-scale computational modelling, where the model is broadly intended to offer a best approximation to a variety of larger scale behaviours being studied. The cognitive architecture is then considered as a system of competences, whose elements are themselves more specific cognitive components. In this way, it is possible to simulate and study the effects not only of specific empirical models, as we do in this article, but also their mutual interaction. Importantly, this approach allows us to study the behaviour that *emerges* from their mutual interaction. Some cognitive architectures are explicitly inspired by biology, and some are more abstract. Some are specified in terms of modules associated with competences, while some specify underlying general capacities on which behaviour of components can be based. The Information Dynamics of Thinking (Wiggins, 2020), which we summarise in Section 3.1, falls into this last category.

### 2.2 Prior Related Research in Modelling Music Perception

To our knowledge, few computational models take the explanatory approach of modelling harmony based on an underlying theory of fundamental perception, in contrast to the more descriptive modelling of musical or musicological function based on empirical observation. These latter models have been surveyed extensively in the literature, and further analysis on our part would yield little new insight. Nevertheless, the descriptive models serve as important reference measurements for work such as ours. They are a strictly necessary first step in modelling research (Wiggins, 2011). Parncutt (2011) gives an exhaustive survey of work on this question up to 2010, and this is supplemented, equally thoroughly, with further survey work by Milne et al. (2015).

Other work exists that aim to model pitch space geometrically, as we do here. For instance, Harte et al. (2006) present a toroidal model with properties similar to the space of Krumhansl and Kessler (1982). Chew (2014) presents a musicological account with a spiral model akin to the pitch spiral of Krumhansl and Shepard (1979) and Shepard (1982). Bernardes et al. (2016) present a multi-level tonal pitch space based on a toroidal geometry combined with tuned rules to combine measurements in the space in order to match the geometry of empirical and musicological pitch spaces. To our knowledge, only two other research programmes attempt to derive the properties of music perception directly from an underlying spectral theory: those of, first, Edward W. Large (Large, 2006; Large et al., 2010; Lerud et al., 2019a) and, second, Andrew Milne (Milne et al., 2010, 2011,2015; Milne and Holland, 2016), and their colleagues.

Large’s work is based in dynamical systems science. It uses explicit hierarchical networks of oscillators that effectively model the lower levels of human auditory function. This convincing work, with its firm connection to oscillatory behaviour, was an important inspiration of the IDyOT model (Wiggins, 2020). Its drawback is that the networks require a very significant amount of computing power to function because running the dynamical systems equations on digital machines can be computationally expensive. We circumvent this problem in our approach. Instead of using an actual dynamic oscillator to represent a resonance, we use the oscillator’s static parameters instead. This not only allows our model to be less demanding computationally, but is also supported by geometrical understanding of the representational spaces. Therefore, we can reason directly *about* the system’s behaviour in terms of the geometry of our perceptual model, whereas Large’s model computes *using* the system’s behaviour. From our perspective, both of these approaches are worthy of continuing research.

Like ours, Milne’s model involves the construction and comparison of spectra to model music perception, and like ours, it is “founded on an important bottom-up component that provides its explanatory power” (Milne et al., 2015, p. 368). Both models proceed methodologically from the psychoacoustic assumption that the auditory system perceives similarity between sounds according to their spectral similarity, and then from the musicological assumption that perception of musical sounds are intimately related to corresponding fully harmonic complex tones (Milne, 2013). The key difference between Milne’s model and ours is how spectra are represented, and this, as we will argue in Section 3.1.2, is important, both from the perspective of knowledge representation, and in terms of what the respective models are capable of explaining. Milne represents spectra as vectors in a high dimensional pitch class space, whereas we represent spectra as sums of resonances in (infinite dimensional) function space. From a mathematical perspective there is a very close correspondence: both models can be viewed as using the geometry of the *L*^2^ or *ℓ*^2^ Hilbert space to measure the similarity between functions (signals and spectra). The relevance of the choice of representation to the explanatory nature of these models can be understood as follows. Milne’s model and others like it could be said to provide a partial explanation of *why* music is perceived a certain way: the spectrum of a sound is highly significant to the auditory system. On the other hand, our model attempts a different kind of explanation: *how*. That is, it proposes to explain how the auditory system uses those spectra during the perception of music. Our aim is to model the same musical phenomena using structures which, while as simple as possible, could be mapped to corresponding measurable physiological events in the brain. This is why our model builds from the notion of the resonance as a primitive, which we will see in Section 3.2.

## 3 Theoretical Framework

### 3.1 The Information Dynamics of Thinking

#### 3.1.1 Overview and Hypotheses

The Information Dynamics of Thinking (IDyOT) is a cognitive architecture described in more detail by Wiggins and Forth (2015), Wiggins and Sanjekdar (2019) and Wiggins (2012,2020). In the current paper, we discuss not the architecture itself, but the system of information representation over which it will work. In keeping with terminology from Artificial Intelligence, we refer to this system as a *knowledge representation* (e.g., Brachman and Levesque, 1992): a mathematical system that is capable of denoting and using information at a semantic level, so that the operations available encode and operate on its meaning. The key property of such a system that makes it useful is the capacity to render knowledge explicit which was only implicit in its input, the clearest example being logical deduction of new facts from existing facts and rules.

The contribution of the current article, therefore, is to position this perceptual model of musical knowledge at the core of our larger hypothetical model of cognition, IDyOT. From this systems-level perspective, the central hypothesis of our project concerns the representation of perceptual and cognitive activity in the brain as a matter of *wave shapes*, whose spectral structure determine their meaning relative to other waves. We state our central hypothesis in a strong and a weak form:

**Weak** Brain representations can be described using hierarchically structured Hilbert spaces.

**Strong** Brains structurally and hierarchically represent information using wave shapes produced by neural oscillators implemented in wetware.

In particular, we introduce a novel approach, called *spectral knowledge representation*, which is based on the dynamic properties of waves. We explain this in the next section.

#### 3.1.2 Spectral Knowledge Representation

Knowledge representation and reasoning was one of the earliest subdisciplines of AI (Brachman and Levesque, 1985; 1992). Initially working from an abstract level, it aimed to simulate the ability of the intelligent mind to formulate and reason with concepts, and with rules specified in terms of those concepts. Many initial attempts were based in formal logic. Over the intervening years, emphasis has changed to the learning of concepts and rules from data, either in symbolic form (e.g., Muggleton, 1991) or as encodings in connectionist systems, also known as artificial neural networks (ANNs: Rumelhart and McClelland, 1986; Mehotra et al., 1996). In both cases, it is convenient to represent these concepts and rules as sets of points, regions, functions, or operators in multi-dimensional spaces whose dimensions correspond with features of the world or with its perception by humans (Gärdenfors, 2000).

Within this framework, the current article describes a method of constructing and comparing resonances in order to model perception and cognition, validated using existing empirical results from music perception research. In particular, we also use specific spectra to represent musical concepts and inner product spaces to model similarity and distance. What distinguishes our approach is the way spectra are represented. Mathematically, there are infinitely many, algebraically equivalent, ways to represent a spectrum in Hilbert space. The choice of one particular representation conveys important information about the meaning of the data and the intent of the representer.

With this in mind, we first present *resonances* in Section 3.2.1 as the primitives for spectral knowledge representation. The intention of spectral knowledge representation is not only to model perception and cognition algebraically, but to do so using structures which can be mapped to underlying physiological phenomena. It must therefore start from a set of primitives which capture some minimal unit of neural activity, so we motivate our use of resonances in Section 3.2.2, after which we provide modes of combination and aggregation which capture higher-level perceptual and cognitive phenomena in Section 3.3. Thus, by choosing the resonance representation, we are moving towards a more explanatory model. We justify this claim in Section 3.3.3.

### 3.2 Resonance Representation

#### 3.2.1 Discrete Resonance Spectrum

**Figure 1:**
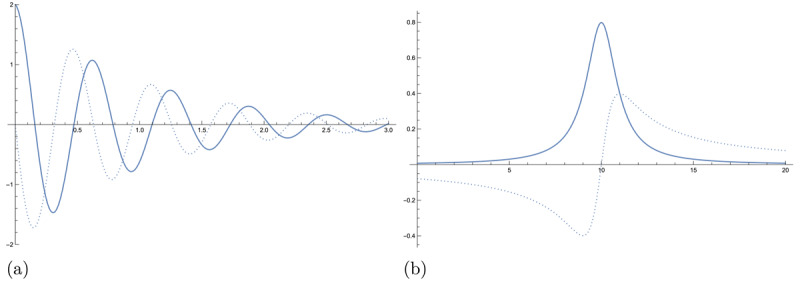
An example of a single resonance in the time domain \[x(t) = 2{e^{ - i(10 - i)t}}\] and frequency domain \[f(\phi) = {\textstyle{i \over {\sqrt {2\pi } }}}({\textstyle{2 \over {\phi - (10 - i)}}})\]. The real part is shown as a solid line and the imaginary part is shown as the dotted line. **(a)** A resonance in the time domain is a complexvalued damped oscillator. **(b)** A resonance in the frequency domain is a complexvalued Lorentzian peak.


A time-domain signal *x(t)* with compact support can be represented as a linear combination of complex oscillators, called *resonances*, defined by Equation 1 and illustrated in Figure 1a.

1 \[x(t) = \sum\limits_{k = 1}^K {{d_k}} {e^{ - i{\omega _k}t}} = \sum\limits_{k = 1}^K | {\mathrm{ }}{d_k}|{e^{i{\psi _k}}}{e^{ - i({\phi _k} + i{\gamma _k})t}}\quad (0 \le t \le T)\]

The Fourier transform^3^ of this compact signal is a frequency-domain function *f*(*ϕ*) called the *resonance spectrum*. The spectrum is represented as a set of Lorentzian peaks^4^ with residues *d_k_* and locations *ω_k_* in Equation 2 and illustrated in Figure 1b.


2 \[f(\phi) = \frac{i}{{\sqrt {2\pi } }}\sum\limits_{k = 1}^K {\frac{{{d_k}}}{{\phi - {\omega _k}}}} = \frac{i}{{\sqrt {2\pi } }}\sum\limits_{k = 1}^K {\frac{{|{d_k}|{e^{i{\psi _k}}}}}{{\phi - ({\phi _k} + i{\gamma _k})}}} \]

Each of the *K* resonances that comprise the signal/spectrum are defined by a pair of complex spectral parameters: a complex resonant amplitude *d_k_* and complex resonant frequency *ω_k_*. It is often convenient to express these two complex parameters as four real-valued spectral parameters, |*d_k_*|, *ψ_k_, ϕ_k_* and *γ_k_*, such that \[{d_k} = \;|{d_k}|{e^{i{\psi _k}}}\] and *ω_k_* = *ϕ_k_* + *iγ_k_*. These spectral parameters have the following interpretation:

**Amplitude |*d_k_*|:** The modulus of the complex amplitude *d_k_*. In the time domain, it is the initial amplitude of the oscillator. In the frequency domain, it contributes to the height of the resonance peak on the frequency axis.

**Phase *ψ_k_*:** The argument of the complex amplitude *d_k_*. In the time domain, it is the initial phase of the oscillator. In frequency domain, it has the effect of rotating the complex plane about the resonance peak.

**Frequency *ϕ_k_*:** The real part of the complex frequency *ω_k_*. In the time domain, it is the rate of oscillation of the resonance. In the frequency domain, it determines the center of the resonance peak along the frequency axis.

**Decay *γ_k_*:** The imaginary part of the complex frequency *ω_k_*. In the time domain, it is the rate of decay of the oscillator. In the frequency domain, it determines the polarity of the resonance peak and contributes to its width on the frequency axis.

#### 3.2.2 Modeling Neural Dynamics with Resonances

Cognitive science has, over the past two decades, moved to embrace paradigms which are less discrete and logical than its original computational metaphor. For example, (Spivey, 2008) gives a convincing account of how a continous analogue system, such as a brain, can perform cognitive computations which seem superficially to be those of a discrete system. *Attractor states* in the state space of a smooth dynamical system pull the state of the system towards themselves, requiring significant input of an appropriate kind to cause a change, much as a light switch uses a spring to hold it in whichever position it is put. Large (2006) takes the dynamical systems argument to a more perceptual level, and connects it with general auditory perception and even general learning (Large et al., 2010). Friston (2010) models cognition as a whole in terms of a dynamical system whose aim is to minimise the physical concept of *free energy*. Our work is framed by broadly the same mathematical approach.

In this vein, neural dynamics can be formulated in a general way with a (stochastic) differential equation (Friston, 2011) that describes the flow of neural states *x* according to some function *f* that is influenced by external inputs *u* and random noise introduced by the *ϵ_x_* term.


3 \[x' = f(x,u) + {\epsilon_x}\]

Since we do not generally have direct access to these neural states, we can instead measure *y*, an observable manifestation of those states governed some function *h*, again influenced by external inputs *u* with random noise in the measurement introduced by *ϵ_y_*.


4 \[y = h(x,u) + {\epsilon_y}\]

These neural states can refer to individual synaptic organization or population-level neural assemblies depending on the domain of the problem of interest (Large et al., 2010; Friston et al., 2014; Hoppensteadt and Izhikevich, 1996; Lerud et al., 2019b). In this formulation, Equations 3 and 4 must capture the complicated nonlinear relationships present in a neural system according to connectivity of different neural assemblies. Depending on the structure of *f*, these sorts of non-linear differential equations often do not admit a closed-form solution; however, we have a way in. The Hartman-Grobman theorem (Strogatz, 2015) states that, for a given nonlinear dynamical system *x*’ = *f*(*x*), near a stationary point *f*(*x̃*) = 0, the system is equivalent to its local linearization according to the Jacobian at that stationary point *J_f_*(*x̃*) = *A*, where the (*i,j*)-th entry of *J_f_*(*x*) = *∂f_i_ /∂x_j_*.


5 \[x' = f(x) \approx Ax\]

So despite the non-linearities present in the system, we can still represent the dynamics linearly, granted we are near a stationary point. This allows us to write Equation 3 as follows, ignoring the external inputs and noise for now. Given an initial state *x*(0), we can solve for *x* with the matrix exponential.


6 \[x' = Ax \Rightarrow x = {e^{At}}x(0)\]

Decomposing *A* according to the spectral theorem *A* = *U*ΛU*, where Λ is a diagonal matrix of eigenvalues, and *U* is a unitary matrix of orthonormal eigenvectors, we have


7 \[x = {e^{tU\Lambda {U^*}}}x(0) = U{e^{t\Lambda }}{U^*}x(0) = \sum\limits_k {{e^{{\lambda _k}t}}} {P_k}x(0) = \sum\limits_k {{e^{{\lambda _k}t}}} {x_k}\]

where *P_k_* is the projector onto the *k*th eigenstate, making *x_k_* = *P_k_x*(0) the projection of the initial state *x*(0) onto the *k*th eigenstate. Assuming that *h* can also be linearized at the stationary point with coefficient (row) vector b*, we have


8 \[y = {b^*}x = \sum\limits_k {{e^{{\lambda _k}t}}} {b^*}{x_k} = \sum\limits_k {{b_k}} {e^{{\lambda _k}t}}\]

Rewriting a few terms to be in line with Equation 1, i.e., *λ_k_* = *–iω_k_, b_k_* = *d_k_*, we arrive at the resonance representation.


9 \[x(t) = \sum\limits_{k = 1}^K {{d_k}} {e^{ - i{\omega _k}t}}\]

This is all to say, if you accept that neural dynamics can be modeled as a dynamical system as in Equation 3, then resonance representations are a necessary consequence of that modelling. In particular, resonances describe the linear behaviour of the dynamical system near a stationary point. As long as we are near that point, the previously ignored terms (external input and noise) will either also be linear and so take the form of resonances, or the non-linearities will have a small effect on the dynamics of the system in comparison to its linearization. As such, we will continue to omit these non-linearities in the following analysis. This is not to say that the non-linearities do not exist or do not matter, but that resonances are a necessary part of the equation no matter what those non-linearities look like.

### 3.3 Resonance Space

#### 3.3.1 Hilbert Space of Resonances

Intuitively, the inner product ⟨*f*|*g*⟩ between two resonance spectra, *f*(*ϕ*) and *g*(*ϕ*), measures the extent to which the resonances of *f* match with the resonances of *g*. The inner product can be considered as the scaled projection from *f* onto *g*, so when the resonances of the two spectra are distributed similarly, the magnitude of their inner product will be large. If the two spectra have very different structure, the magnitude of their inner product will be small. Note that here, we take care to say the *magnitude* of the inner product, since generally, the inner product is a complex-valued quantity.

Formally, we take the *L*^2^ inner product between each of the *J* resonances of spectrum *f* with each of the *K* resonances of spectrum *g* as follows in Equation 10.


10 \[\begin{array}{*{20}{l}}
{\left\langle {f|g} \right\rangle }&{ = \int_{ - \infty }^\infty {\;\left({\frac{i}{{\sqrt {2\pi } }}\sum\limits_{j = 1}^J {\frac{{|{d_j}|{e^{i{\psi _j}}}}}{{\phi - ({\phi _j} + i{\gamma _j})}}} } \right){\mathrm{ }}\left({\frac{i}{{\sqrt {2\pi } }}\sum\limits_{k = 1}^K {\frac{{|{d_k}|{e^{i{\psi _k}}}}}{{\phi - ({\phi _k} + i{\gamma _k})}}} } \right) * d\phi } }\\
{}&{ = \sum\limits_{j = 1}^J {\sum\limits_{k = 1}^K {\frac{{i|{d_j}||{d_k}|{e^{i({\psi _j} - {\psi _k})}}}}{{({\phi _j} - {\phi _k}) + i({\gamma _j} + {\gamma _k})}}} } {\mathop{\rm sgn}} ({\gamma _j})}
\end{array}\]

where sgn(*γ_j_*) = sgn(*γ_k_*) and ⟨*f*|*g*⟩ = 0 if sgn(*γ_j_*) ≠ sgn(*γ_k_*) and postfix * denotes the complex conjugate. This implies that resonances with decay of opposite sign are always orthogonal, since their inner product is 0. In the remainder, we will omit mention of these orthogonal terms, and we will assume that *γ_j_* > 0, *γ_k_* > 0 to avoid a profusion of sgn(*γ_k_*)’s throughout our expressions. These expressions can simply be negated if *γ_j_* < 0, *γ_k_* < 0.

The numerator inside the summation of Equation 10 accounts for the amplitude and phase of resonance *j* and *k* of spectra *f* and *g* respectively. When the amplitudes are large and in phase, the numerator will also be large. If those resonances are out of phase, then this term will be smaller. The denominator accounts for the difference in frequency and decay of two constituent resonances. When the frequencies and decays are close, then the denominator will be small, meaning the entire term will be large. If the frequencies and decays are far apart, the denominator will be large, meaning the entire term will be small. Therefore, the inner product between two resonance spectra agrees with our intuitive sense of similarity between them, since resonances with similar spectral parameters will have a large inner product.

The inner product induces a norm \[\parallel f\parallel = \sqrt {\left\langle {f|f} \right\rangle }\], which can be thought of as the length of the resonance spectrum *f* in the Hilbert space of resonances. This is equivalent to the power spectral density of the resonance spectrum. Note that in equation 11, both summations are from 1 to *K*, indicating that resonances *k* and *k*’ are taken from the same spectrum *f*.


11 \[\parallel f{\parallel ^2} = \sum\limits_{k = 1}^K {\sum\limits_{k' = 1}^K {\frac{{i{d_k}d_{k'}^*}}{{{\omega _k} - \omega _{k'}^*}}} } = \sum\limits_{k = 1}^K {\sum\limits_{k' = 1}^K {\frac{{i|{d_k}||{d_{k'}}|{e^{i({\psi _k} - {\psi _{k'}})}}}}{{({\phi _k} - {\phi _{k'}}) + i({\gamma _k} + {\gamma _{k'}})}}} } \]

To account for potential differences in amplitudes between spectra, it is common to normalize the inner product by using the cosine of the angle between *f* and *g*, resulting in the cosine similarity between the two spectra shown in Equation 12. Milne et al. (2011, Sec. 5) provide a more in-depth analysis of other spectral similarity measures. Thus, if *f* and *g* are pointing in almost the same direction in resonance space, then this quantity will be close to one; if they are nearly orthogonal, then it will be close to zero.


12 \[{s_c}(f,g) = \cos \theta = \frac{{{\mathrm{Re}}\left[ {\left\langle {f|g} \right\rangle } \right]}}{{\left\| f \right\|{\mathrm{ }}\left\| g \right\|}}\]

If a measure of distance is required instead of a similarity, the cosine similarity can be converted to a cosine distance^5^ as in Equation 13.


13 \[{d_c}(f,g) = 1 - {s_c}(f,g) = 1 - \frac{{{\mathop{\rm Re}\nolimits} \left[ {\left\langle {f|g} \right\rangle } \right]}}{{\left\| f \right\|{\mathrm{ }}\left\| g \right\|}}\]

#### 3.3.2 Representing Functions with the Resonance Basis

Suppose we have a continuous function *f* over a real closed interval [*a, b*]. The Weierstrass approximation theorem states that there exists a polynomial *p_N_* of degree *N* that uniformly approximates *f* to arbitrary precision *ϵ* > 0; i.e., ‖*f* – *p_N_*‖*_∞_* < ϵ and lim*_N_*_→__∞_ ‖*f* – *p_N_*‖_∞_ = 0, where ‖∙‖*_∞_* denotes the supremum norm. So for a desired precision *ϵ* > 0, there is always a finite degree polynomial *p_N_* that well approximates *f*.

The Padé approximant of a function is a rational function whose Maclaurin series expansion agrees with the series expansion of that function for the first *J* + *K* terms, where *J* and *K* are the degrees of the numerator and denominator polynomials respectively. For *ϵ* > 0, *p_N_* has finitely many terms (*N* < *∞*) and is equal to its Maclaurin series expansion. Therefore, the Padé approximant of *p_N_*, with *J* + *K* = *N*, is exact for a fixed precision *ϵ* > 0. The poles of the Padé approximant of *p_N_* correspond to the resonant frequencies *ω_k_* and the residues at those poles correspond to resonant amplitudes *d_k_*. Therefore, the continuous function *f* can be approximated to arbitrary precision *ϵ* > 0 with *K* resonances. For certain functions, perfect precision *ϵ* = 0 is possible with only finite *K*. In general, the number of resonances *K* depends both on the function *f* and desired precision *ϵ*, so the uniqueness of the resonance representation is also dependent on the chosen precision *ϵ*.

When we have only a finite number of *N* distinct points (*x_n_, f*(*x_n_*)), there exists a unique polynomial of lowest degree ≤ *N-*1 that interpolates those points exactly, called the Lagrange interpolating polynomial. With that polynomial in hand, we can again use the Padé approximant to find the associated resonant amplitudes and frequencies of the resonances. If a bit of error (*ϵ* > 0) is acceptable in interpolating those points, then a polynomial of even lower degree can be found, for instance using least squares approximation. In practice, we are often interested in analyzing a set of discrete data points, so the uniqueness of the Lagrange interpolating polynomial ensures the uniqueness of the resonance representation for those data.

Now knowing that it is always possible to represent a function with resonances, suppose that a signal *f* ∈ *L*^2^ can be represented as a combination of *K* resonances *r_k_*.


14 \[f(t) = \sum\limits_{k = 1}^K {{d_k}} {e^{ - i{\omega _k}t}} = \sum\limits_{k = 1}^K {{d_k}} {r_k}(t)\]

Given an orthonormal basis (*ϕ_n_*) in *L^2^*, the function *f* has a unique representation.


15 \[f = \sum\limits_{n = 1}^\infty {\left\langle {f|{\varphi _n}} \right\rangle } {\mathrm{ }}{\varphi _n}\]

Since the inner product is linear in its first argument, and swapping the summations, we have


16 \[f = \sum\limits_{n = 1}^\infty {\left\langle {\sum\limits_{k = 1}^K {{d_k}} {r_k}|{\varphi _n}} \right\rangle } {\varphi _n} = \sum\limits_{n = 1}^\infty {\sum\limits_{k = 1}^K {{d_k}} } \left\langle {{r_k}|{\varphi _n}} \right\rangle {\varphi _n} = \sum\limits_{k = 1}^K {{d_k}} \sum\limits_{n = 1}^\infty {\left\langle {{r_k}|{\varphi _n}} \right\rangle } {\varphi _n}\]

Therefore, each resonance *r_k_* is represented by a linear combination of infinitely many orthonormal bases *ϕ_n_*,


17 \[{r_k} = \sum\limits_{n = 1}^\infty {\left\langle {{r_k}|{\varphi _n}} \right\rangle } {\varphi _n}\]

and each coefficient ⟨*f*|*ϕ_n_*⟩ is represented as a weighted sum of projections onto the resonances.


18 \[\left\langle {f|{\varphi _n}} \right\rangle = \sum\limits_{k = 1}^K {{d_k}} \left\langle {{r_k}|{\varphi _n}} \right\rangle \]

This means that we can translate at will between a resonance representation and an orthonormal representation as long as we know the inner product ⟨*r_k_*|*ϕ_n_*⟩; i.e., the projection of a resonance onto an orthonormal base.

For example, consider the Fourier basis^6^ in *L*^2^(*-π,π*), which looks like


19 \[{\varphi _n}(t) = \frac{{{e^{ - int}}}}{{\sqrt {2\pi } }}\]

The projection of resonance *r_k_* onto Fourier base ϕ*_n_* then takes the form


20 \[\left\langle {{r_k}|{\varphi _n}} \right\rangle = \int_{ - \pi }^\pi {{e^{ - i{\omega _k}t}}} \frac{{{e^{int}}}}{{\sqrt {2\pi } }}dt = \sqrt {2\pi } {\mathop{\rm sinc}\nolimits} (n - {\omega _k})\]

where sinc(*x*) = sin(πx)/(πx) is the normalized sinc function. Therefore we can represent a resonance *r_k_* as


21 \[{r_k} = \sum\limits_{n = - \infty }^\infty {{\mathop{\rm sinc}\nolimits} } (n - {\omega _k}){e^{ - int}}\]

Here, each resonance corresponds to an infinite sum of non-decaying complex oscillators each weighted by a translated, normalized sinc function. Since in general the imaginary part of each *ω_k_* is non-zero, each coefficient must be non-zero, so none of the terms drop out, and an infinite number of Fourier base terms are required to represent a single resonance.

By contrast, each Fourier coefficient is a weighted sum of sinc functions involving the *K* pairs of resonance parameters (*d_k_, ω_k_*).


22 \[\left\langle {f|{\varphi _n}} \right\rangle = \sqrt {2\pi } \sum\limits_{k = 1}^K {{d_k}} {\mathop{\rm sinc}\nolimits} (n - {\omega _k})\]

So assuming that a signal can be decomposed into *K* resonances, in order to represent a single resonance in the Fourier basis, we require an infinite number of terms, but conversely, to represent a single Fourier base, we only need *K* resonances. This asymmetry demonstrates how the non-orthogonality of resonances results in a parsimonious representation of the function. Sure, an orthonormal basis is often easier to work with, but in general, you need an infinite number of terms in order represent a function. If the function admits representation with a finite combination of resonances, then the resonance representation will have infinitely fewer terms.

#### 3.3.3 Comparison with the Fourier Basis

Many signal processing techniques have been developed that seek to capture different aspects of a uniformly sampled signal by representing it using different basis functions such as polynomials or wavelets (Mallat, 2009). Among these, the best known technique is the Discrete Fourier Transform (DFT: Oppenheim and Schafer, 2010), generally implemented using the Fast Fourier Transform (FFT: Mallat, 2009). Here, we give a brief comparison of our method with the DFT (Equation 23).


23 \[{\hat f_k} = \frac{1}{{\sqrt N }}\sum\limits_{n = 0}^{N - 1} {{f_n}} {e^{i2\pi k\frac{n}{N}}}\]

The DFT decomposes a signal into a truncated Fourier basis, i.e., a linear combination of non-decaying oscillators whose real-valued frequencies are evenly-spaced and fixed to a grid according to the length of the sampled signal. For example, suppose a signal sampled at 44.1kHz has 1024 samples. The frequency bins^7^ are determined by Eqn. 23 to be located at 0Hz, 43Hz, 86Hz, and so on, regardless of the structure of the signal. By contrast, the resonance spectrum decomposes a signal into a linear combination of driven or damped oscillators, neither whose frequencies are evenly-spaced, nor whose position is fixed according to the number of samples in the signal. The positions of the spectral peaks are chosen according to the structure of the specific signal at hand, placing more resonances where more detail is required and fewer where less is happening.

By constraining the non-decaying oscillators of the DFT to the uniform, fixed grid, they are mutually orthogonal over one period of length *N*. Therefore, the output of the DFT is equivalent to a vector whose dimensions correspond to the frequency of each oscillator. These coefficients capture the overall shape of the spectrum in a uniform way. This is extremely convenient for directly comparing full spectra, as the comparison can be performed purely in terms of the coefficients; however, it has the disadvantage that the exact size and location of peaks in the spectra are not directly represented, and so must be inferred from the coefficients of this global representation. For instance, in our earlier example, suppose the signal was a pure sine wave with frequency 60Hz; the DFT would distribute spectral power not only at nearby 43Hz and 86Hz, but at all the other frequencies in the grid as well. The resonance spectrum, by contrast, directly represents the size and location of spectral peaks. In our example, it would simply place a resonance at 60Hz. The disadvantage of this local precision is that comparing the overall shape of resonance spectra is less straightforward. It cannot be done component-wise because the resonances are not generally orthogonal. The advantage, however, is that the resonance spectrum gives a much more precise account of the oscillatory *building blocks* of a signal, resulting in a parsimonious representation of the data.

Despite the benefits of resonance representations, there are two significant drawbacks that make using them more difficult in practice. First, though the non-orthogonality of resonances allows for a more parsimonious representation of a signal or spectrum due to interference, it also makes manipulating and calculating with resonances more complicated. For instance, when calculating the norm in a Fourier basis with *N* terms, each component can be treated independently of the others, so the resulting sum involves just *N* terms. Since resonances are generally non-orthogonal, the components of a spectrum are not independent, so the resulting sum has *N^2^* terms, evident in the double sum in Equation 11. Whereas in the Fourier basis, or any orthonormal basis, cross-terms always evaluate to zero, this is not the case with resonances, since the cross-terms may contribute a non-trivial amount to the overall result. Second, calculation of resonances from a given signal is not nearly as efficient as the FFT, making them unsuitable for practical applications that require near real-time processing.

It should be noted that as elements of the same *L^2^* function space, for a fixed signal of length *N*, the DFT spectra and resonance spectra are equivalent with respect to the inner product, since the resonances are merely different basis functions — but then, all bases are equivalent in that regard. That is, distances and angles are not affected by the choice of coordinate system. The key difference, as discussed in Section 3.1.2, is how they are represented. We argue that resonances are more useful, and perhaps a more veridical representation of the physical system being modelled in our applications, such as modelling music perception in Section 4. Ultimately, given the resonance representation of a spectrum, calculating the total spectrum is trivial; that is, we can recover the output of the DFT directly. However, going the other way and finding the spectral parameters of resonances from the output of the DFT is more difficult, for instance by solving a nonlinear system in Equation 22. In addition, many techniques used in conjunction with the FFT, such as smoothing and windowing kernels, or that rely on the FFT, such as MFCCs (Davis and Mermelstein, 1980), can be translated to work with resonances instead.

## 4 Empirical Application in Music Perception

### 4.1 Modelling Key Affinity and Inter-Key Distance

#### 4.1.1 Harmonic Operator

Many of the ideas presented in the current paper are inspired by the seminal ideas of the music theorist, Jean Phillippe Rameau (Rameau, 1722; Christensen and Rameau, 1987). Rameau systematically derived all the commonly accepted chords of Western classical harmony, including major, minor, augmented and diminished chords, different inversions, and so on, from the harmonic series: specifically, from the ratios between harmonics 4, 5 and 6 (the major triad), in just intonation. In doing so, he proposed what was effectively a perceptual theory of harmony, based on the relationships between the fundamental frequencies of tones in comparison to those found in their harmonics. Rameau’s emphasis on the major triad is reflected in our use of triads in our harmonic distance model (Section 4.3).

To implement these ideas, we now define a harmonic operator *H* that transforms a resonance spectrum *f* to a harmonic series of resonances called the *harmonic spectrum Hf*.


24 \[Hf \equiv H[f(\phi)]{\mathrm{ }}: = \frac{i}{{\sqrt {2\pi } }}\sum\limits_{k = 1}^K {\sum\limits_{n = 1}^N A } (\phi,n)\frac{{|{d_k}|{e^{in{\psi _k}}}}}{{\phi - (n{\phi _k} + i{\gamma _k})}}\]

This operator is a natural interpretation of a harmonic series of resonances. Having this operator is important because it allows us to refer to harmonic structure without mentioning full spectra. The frequency *ϕ_k_* and phase *ψ_k_* of each of *K* resonances are multiplied by the harmonic number *n* to encode the relationship between the fundamental frequency and the overtones of the series. In addition, the amplitude of each overtone is weighted by an attenuation function *A*(*ϕ,n*) since higher overtones and frequencies are generally of lower power than lower overtones.

**Figure 2:**
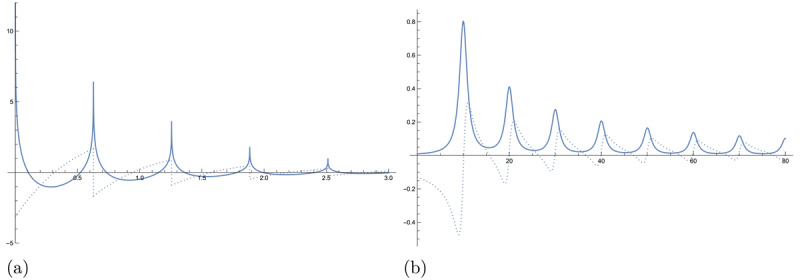
An example of a harmonic signal and spectrum generated from a single seed resonance with |*d_k_*| = 2, *ψ_k_* = 0, *ϕ_k_* = 10, and *γ_k_* = *–*1, the same as shown in Figure 1, with attenuation *A*(*ϕ, n*) = *n*^–1^ and *N* → *∞*. The Re[*Hf*] is shown as the solid line and the Im[*Hf*] is shown as the dotted line. **(a)** A harmonic resonance in the time domain is a complex-valued damped train of spikes. **(b)** A harmonic resonance in the frequency domain is a complex-valued train of Lorentzian peaks.

The choice of attenuation function *A*(*ϕ, n*) has a large impact on the behaviour of the series, especially when the total number of overtones *N* → *∞* since it determines whether the infinite series will converge and thence whether *H* is a bounded or unbounded operator. In general, *A*(*ϕ, n*) can be a function of both frequency *ϕ* and harmonic number *n*, and should be chosen depending on the application in question. For instance, 1*/f* noise is a commonly observed phenomenon in signal processing and statistical physics (Ward and Greenwood, 2007) where the power spectral density decays according to 1*/ϕ*, making it purely a function of frequency *ϕ*. Another possibility is to attenuate according only to the harmonic number, such as *A*(*ϕ, n*) = *n*^–1^, as shown in Figure 2.

The inner product ⟨*f*|*Hg*⟩ between a resonance spectrum *f* and a harmonic spectrum *Hg*, shown in Equation 25, measures the extent to which the distribution of power of a resonance spectrum coincides with the distribution of power of a harmonic spectrum. The more the resonance spectrum coincides with the overtones of the harmonic spectrum, the larger this inner product will be. Therefore, this inner product measures the relative harmonicity of a given resonance spectrum with respect to another candidate spectrum. The relative harmonicity can then be used to calculate summary measures, such as the maximum (Milne, 2013) or entropy (Harrison and Pearce, 2018) of harmonicity across all candidates in some set of spectra, e.g., the set of all translations of some spectrum. As a special case, if the harmonic spectrum is generated from a single seed frequency, then this inner product measures the extent to which that frequency corresponds to the fundamental frequency of the resonance spectrum *f*.


25 \[\left\langle {f|Hg} \right\rangle = i\sum\limits_{n = 1}^N A (\phi,n)\sum\limits_{j = 1}^J {\sum\limits_{k = 1}^K {\frac{{|{d_j}||{d_k}|{e^{i({\psi _j} - n{\psi _k})}}}}{{({\phi _j} - n{\phi _k}) + i({\gamma _j} + {\gamma _k})}}} } \]

The inner product between two harmonic spectra, ⟨*Hf*|*Hg*⟩, measures the extent to which the two harmonic spectra overlap. For example, suppose that the two resonance spectra *f* and *g* have most of their power concentrated at the frequencies *ϕ_j_* and *ϕ_k_* respectively. When *ϕ_j_* is an overtone of *ϕ_k_*, all the higher overtones will also be aligned, meaning their inner product will be large. If they are not in a harmonic relationship and their higher overtones do not generally align, then the inner product will be smaller.


26 \[\left\langle {Hf|Hg} \right\rangle = i\sum\limits_{n = 1}^N {\sum\limits_{m = 1}^N A } (\phi,n)A(\phi,m)\sum\limits_{j = 1}^J {\sum\limits_{k = 1}^K {\frac{{|{d_j}||{d_k}|{e^{i(n{\psi _j} - m{\psi _k})}}}}{{(n{\phi _j} - m{\phi _k}) + i({\gamma _j} + {\gamma _k})}}} } \]

The cosine similarity and cosine distance between harmonic spectra is defined analogously as with resonance spectra, shown in Equations 12 and 13.

#### 4.1.2 A Note on Terminology and Notation

In describing this work, we find a confusing overloading of the word *harmonic*, due to its various uses in mathematics, signal analysis, and musicology. In mathematics, the term “harmonic” may refer to harmonic analysis, i.e., the decomposition of a function into its spectral components, or the harmonic series \[\Sigma _{n = 1}^\infty {\textstyle{1 \over n}}\]. Closely related, in signal processing, a harmonic is a frequency that is an integer multiple of some fundamental frequency. By contrast, in musicology the harmonic function of pitch, for example, refers to how it contributes to musical harmony in the context of a key. Though these definitions are related (and in this paper we demonstrate that this is fundamentally so), to avoid confusion we reserve distinct terminology for each usage.

When referring to a series that decays similarly to the harmonic series 1*/n*, we will say that it is *harmonically attenuated*. When referring to integer multiples of some fundamental frequency, we will refer to *overtones*, though if one frequency is an integer multiple of another, those two frequencies are in a *harmonic relationship*. When discussing harmonic function in the Western musicological sense, we will use *tonal-harmonic function*.

Finally, we frequently use the terms *resonance spectrum* and *harmonic spectrum*. As defined in Sections 3.2.1 and 4.1.1, a resonance spectrum is composed of resonance peaks and a harmonic spectrum is a resonance spectrum that has been operated on by our harmonic operator. It might be the case that some component resonances combine to produce musical harmony; they are still considered a resonance spectrum. It might even be the case that the component frequencies of a resonance spectrum are harmonically related; nonetheless, they are still considered a resonance spectrum. Only when a spectrum has been operated on by the harmonic operator does it, in our terms, become a harmonic spectrum. In short, the structure of a spectrum may be harmonic, musical or otherwise, but that plays no role in its designation as a resonance spectrum or harmonic spectrum. In our terminology, it is *only* the application of our harmonic operator to a resonance spectrum that designates it as a harmonic spectrum.

A comparable overloading of note names and function names occurs between music and mathematics. We use the mathematical function names *f* and *g* extensively in this paper, because to do otherwise would be unnatural from a mathematician’s perspective. These symbols are distinct from the musical tones of the same names, denoted F and G, respectively.

#### 4.1.3 Relating Fundamental and Full Spectra

The primary motivation for developing the mathematics of the harmonic operator and inner product in Section 3.2 is to formalize the notion that two tones^8^ are perceptually similar when their overtones coincide, *even if those overtones are not actually present in the signal*. Using the terminology defined above, this means that perceived similarity between two tones is measured by the coincidence of their corresponding harmonic spectra, which is achieved by taking the inner product between their harmonic spectra. This insight entails a range of opportunities in modelling of music perception, each of which adds support to operations on the resonance space as a model of neural activity during the corresponding task, which we demonstrate in Section 4.2 and Section 4.3.

In practical terms, the harmonic operator adds in extra overtones to each tone or chord in order to construct the harmonic spectrum. To be clear, these overtones are added for the full spectrum of the tone/chord, not just for the fundamentals of that spectrum, though in the following empirical portion we focus on fundamental spectra; however, since the harmonic operator is a linear operator over resonances, there is a linear relationship between the harmonic spectrum of the fundamental spectrum (i.e., the spectrum consisting only of the fundamental frequencies, and not the overtones) and the full spectrum of the tone or chord, (i.e., the spectrum consisting of fundamentals, overtones, and non-harmonic content). The linearity of the harmonic operator implies that the set of harmonic operators forms a vector space. That is, we can talk about adding and scaling harmonic structures directly without first referring to the resonance spectra (fundamental, full, or otherwise) to which the harmonic operators are applied.

Given the full spectrum *f* of an acoustic sound, suppose we can split it up into fundamental spectrum *f*_0_, overtones *f_n_*, and non-harmonic content/noise *ϵ*.


27 \[f = {f_0} + \sum\limits_n {{f_n}} + \epsilon \]

Note that when *f* is a polyphonic sound like a chord, *f*_0_ is not a single fundamental, but a set of them, each with their own overtones altogether represented by Σ*_n_ f_n_*. Since the harmonic operator *H* is linear, i.e., *H*[*αf* + *βg*] = *αHf* + *βHg*, we can apply it to each term separately, giving the following.


28 \[Hf = H{f_0} + \sum\limits_n H {f_n} + H\epsilon \]

The frequency *ϕ_n_* of each *f_n_* is an integer multiple of the frequency *ϕ*_0_ of *f*_0_, i.e., *ϕ_n_* = *nϕ*_0_, so not only do overtones added by *H f*_0_ coincide with the natural overtones Σ*_n_ f_n_*, but all the overtones added by each *H f_n_* will coincide in frequency with one another as well. Therefore, *H f* is roughly proportional to *H f*_0_, except with an extra term involving non-harmonic content. If we assume that most of the signal power is contained in the harmonic content, we can ignore the non-harmonic term *ϵ*. Further, when we consider two different harmonic operators *H* and *H̃* with different attenuation functions *A*(*n,ϕ)* and *Ã*(*n,ϕ*) respectively, then the differences between *H f* and *H f*_0_ can be absorbed into the differences between *H* and *H̃*.


29 \[Hf = \tilde H{f_0}\]

In the simplest case, when *H* is the identity operator, we have *f* = *H̃f_0_*, meaning *H̃* takes the fundamental *f*_0_ and generates all the harmonic content found in the given *f*. This means that since we can apply a given harmonic operator *H* to a full spectrum *f*, then we can find an equivalent *H̃* to apply to the fundamental spectrum *f*_0_, to arrive at the same overall harmonic spectrum. Due to this relationship, in the remainder of this article, we will focus on fundamental spectra to demonstrate how resonances, resonance space, and the harmonic operator can be used to model key affinity and inter-key distance, as opposed to using the full spectrum of a tone or chord. This is not to say that the overtones and non-harmonic content present in full spectra do not matter in music perception – in fact, even in Equation 29 the exact relationship between *H* and *H̃* depends on how power is distributed among the overtones of *f* – just that analyzing a fundamental spectrum is a reasonable proxy for analyzing a full spectrum. This methodology allows us to make the implicit harmonic structure of a full spectrum *f* (or harmonic spectrum *H f*) into an explicit relationship between the fundamentals *f*_0_ and overtones *H f*_0_. This separation allows us to generalize across many related sounds that share harmonic structure. For instance, the same instrument playing different tones can be represented by one harmonic operator and the fundamental spectra corresponding of those tones.

#### 4.1.4 Considerations in the Design of the Model

In our model, we can view the spectrum *f* of the musical key as a simulation of the *memory of a key*, and the tone spectrum *g* as a simulation of the *experience of the tone*. That is to say, we think of the spectrum of a musical key as an oscillatory circuit which may be stimulated to some degree by the tone oscillation, thus triggering the experience of the key. It is also important to understand that there is a principle here, borrowed from Rameau (1722), and also arising in the terminology of Krumhansl and Kessler (1982) and Parncutt (2011): a triad is sufficient to *define* a key. We are inclined to follow this principle; the reader who disagrees may prefer to call the following sections “chord affinity” and “triad distance” instead.

Since we propose the key is defined by the triad, and the triad is defined by the fundamental frequencies of its pitches, we represent the key triad as a resonance spectrum *f* as consisting only of fundamental frequencies *ϕ*_1_, *ϕ*_2_, and *ϕ*_3_, in a 12 tone equal-tempered scale, shown in Equation 30. The amplitude of each pitch in the triad is |*d*_1_| = 1, 0 ≤ |*d*_2_| ≤ 1, 0 ≤ |*d*_3_| ≤ 1 and each initial phase *ψ*_1_ = *ψ*_2_ = *ψ*_3_ = 0.


30 \[f(\phi) = \frac{i}{{\sqrt {2\pi } }}\left({\frac{1}{{\phi - ({\phi _1} + i{\gamma _0})}} + \frac{{|{d_2}|}}{{\phi - ({\phi _2} + i{\gamma _0})}} + \frac{{|{d_3}|}}{{\phi - ({\phi _3} + i{\gamma _0})}}} \right)\]

We begin with a parameterized form of harmonic attenuation shown in Equation 31:


31 \[A(\phi,n) = \frac{1}{{{n^\alpha } + \beta }}\]

where *α* > 0 controls the rate of attenuation with respect to the harmonic number *n* and *β* ∈ ℝ flattens the overall shape. This specific sort of harmonic attenuation is chosen due to the relationship between overtones proposed by Klapuri (2006), who observed a 1*/n* relationship when fitting a fundamental frequency salience function to recordings of musical instruments in a polyphonic context. A similar approach of harmonically attenuation using *n^–^*^ρ^(*ρ* > 0) is explored by Milne et al. (2011, ff.).

This gives the following form of the harmonic spectrum of a triad *Hf*, with parameters *γ*_0_, *N, α, β*, |*d*_2_|, |*d*_3_| shown in Equation 32.


32 \[Hf(\phi) = \frac{i}{{\sqrt {2\pi } }}\sum\limits_{n = 1}^N {\frac{1}{{{n^\alpha } + \beta }}} \left({\frac{1}{{\phi - (n{\phi _1} + i{\gamma _0})}} + \frac{{|{d_2}|}}{{\phi - (n{\phi _2} + i{\gamma _0})}} + \frac{{|{d_3}|}}{{\phi - (n{\phi _3} + i{\gamma _0})}}} \right)\]

**Figure 3:**
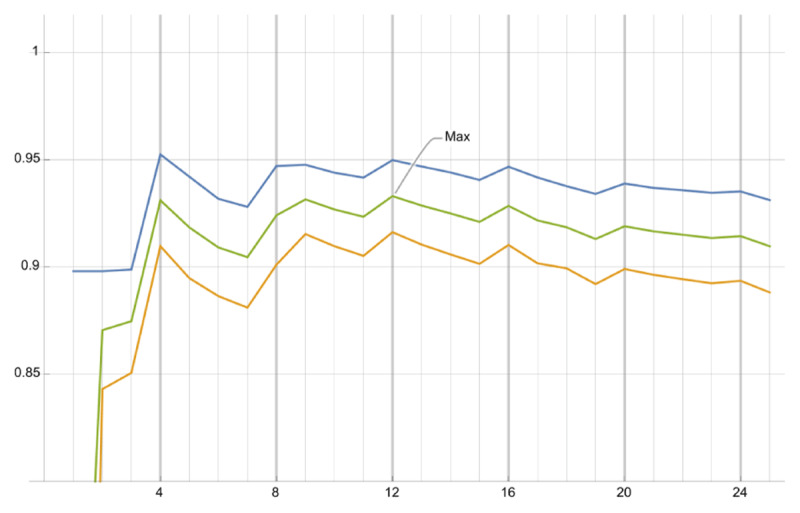
Correlation of best fit models over a range of *N*, the total number of overtones in the harmonic spectrum. The blue line is fit using the key affinity profile data. The orange line is fit using the inter-key distance profile data, and the green line is the mean of those two curves. The maximum correlation occurs at *N* = 12, with a close second at *N* = 4.

Similarly to other models in the literature (Milne et al., 2015, provides a thorough survey), we fit the parameters of our model in Equation 32 to the major and minor key profile data of Krumhansl and Kessler (1982) and separately to the inter-chord distance data from the same paper, including scale and location parameters that do not affect correlation. The parameters were fit to the data for a variety of different values of *N*, the number of overtones generated in the harmonic spectra, ranging from 1 to 25 (see Figure 3). Interestingly, regardless of the value of *N*, the best fit parameterization of the attenuation function effectively resulted in a constant function, i.e., *A*(*ϕ,n*) = *c* for *c* ∈ ℝ, meaning that the overtones generated by the harmonic operator are the same amplitude as their seed resonances.

This flat model only makes sense for finite *N*, so we propose a simplified model with constant attenuation *A*(*ϕ,n*) = 1 to investigate the effect of the total number of overtones *N* on key affinity and inter-key distance (discussed in Sections 4.2 and 4.3). By considering the number of overtones *N* as a free parameter and fitting the finite flat model to the data, we observe the maximum correlation occurs at *N* = 12, with a close second at *N* = 4, shown in Figure 3. In the figure, we observe a pattern with period 4 of local maxima. This likely occurs because all the pitches of the major triad and two of the pitches of the minor triad are (nearly) integer multiples of the tonic at *n* = 4, and therefore all higher integer multiples of 4. When the harmonic spectrum of a given pitch or triad happens to place an overtone at one of these positions, there is a nearly perfect coincidence with the harmonic spectra of five of the six pitches of the major and minor triads. Therefore, in our model, the cosine similarity between their harmonic spectra will be high. Since we see local maxima of the best fit models at these points, something similar is occurring in the data. A similar approach is used by Milne et al. (2015).

Note that if we had used the full spectrum of some chosen acoustic sound to model the triad, we would have fit different parameter values for the model, but the overall effect would be the same, as we argue in 4.1.3. In summary, the model used in the remainder has the form of Equation 33, leaving free parameters *γ*_0_, |*d*_2_|, and |*d*_3_|.


33 \[Hf(\phi) = \frac{i}{{\sqrt {2\pi } }}\sum\limits_{n = 1}^{12} {\left({\frac{1}{{\phi - (n{\phi _1} + i{\gamma _0})}} + \frac{{|{d_2}|}}{{\phi - (n{\phi _2} + i{\gamma _0})}} + \frac{{|{d_3}|}}{{\phi - (n{\phi _3} + i{\gamma _0})}}} \right)} \]

#### 4.1.5 Relation to Brain Structure

From the perspective of modelling, we view resonances as a proxy^9^ for the input information made available to auditory cortex by the human ear. Similar oscillations are produced by successful cochlear/auditory simulations (Lindeberg and Friberg, 2015; Lerud et al., 2019a) and seem to correspond with empirical observation of the operation of neurons (Weiss, 1996) and the effective connectivity of the brain connectome (Friston et al., 2014). At their initial stage, these simulations seek to model the behaviour of the Organ of Corti, which may be thought of as consisting of an array of oscillators of various frequencies that are capable of resonating with components of sounds of the same frequencies. Such a resonance produces a neural oscillation, damped over quite a short time, but of the same frequency as the sound component that stimulated it. Continuous sounds are then represented by series of such oscillations. This view stands in contrast to the previous place theory of pitch perception (Helmholtz, 1954), which held that the position of the oscillation on the Organ of Corti was what determined the sensation of pitch. The importance of the presence of the actual oscillation in the representation is underlined by recent work suggesting that a simulation of the entire perceived sound is reconstructed in the brain stem (Skoe and Kraus, 2010; Kraus and Nicol, 2019).

The basic operation of the harmonic operator entails a certain mathematical structure. An interesting question to ask is whether that structure elucidates, or at least motivates, thinking about potential neural implementations of the same functions. At a very high level, the dynamics of the brain adapt to the dynamics of the environment, which implies that in the case of sound, the resonances corresponding to those dynamics become strongly tuned to the spectral structure of natural sounds from the environment. These sounds often exhibit harmonic structure as a consequence of physical mechanics, so the neural dynamics of the brain adapt to reflect this structure. We speculate that this tuned structure may be thought of as modelling tonotopic maps (Saenz and Langers, 2014). Our harmonic operator can then be thought of as connecting elements of that tonotopic map together, such that harmonic multiples of the resonant frequencies augment each other’s activation, resulting in the sensation of the fused tone (as opposed to the separate overtones). The inner product may be implemented as a combination of addition and multiplication, both of which are possible in neurons (Groschner et al., 2022).

Given representations of musical sounds in the resonance space – that is, assuming we have input to the auditory nerve and cortex in the form of sets of resonances from the ear – we are able to simulate the empirical musical properties explained in the rest of this section.

### 4.2 Key Affinity

Key affinity measures how well a given pitch fits in the context of a key. Pitches that play an important role in the tonality of a key have high affinity for that key, whereas pitches that are perceived as dissonant in the context of a key would have low affinity for that key. Thus, the tones of the tonic triad of the key have high affinity since they are maximally consonant in this context, while the tritone (for example, F♯, in C major) is maximally dissonant and has low affinity with the tonic triad.

In order to measure the affinity of a pitch for a key, we associate a resonance spectrum *f*, composed of resonances with frequencies equal to the tonic triad of the key, and associate another resonance spectrum *g*, composed of a resonance with frequency equal to the fundamental frequency of the pitch. The cosine similarity between the harmonic spectra of *f* and *g*, i.e., *s_c_*(*Hf, Hg*), then represents how well the overtones of the pitch and triad align, which we take to be a measure of key affinity, similar to the method used in Milne et al. (2015).

**Figure 4:**
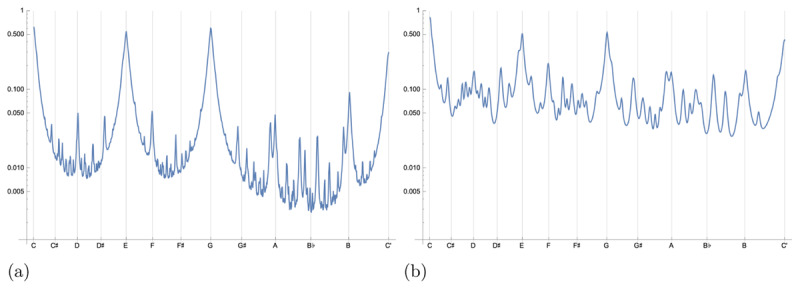
Key affinity *s_c_*(*Hf, Hg*) profiles between the C-major key and all frequencies ranging from C to C’, the octave above. Each equal-tempered semitone is located on the horizontal axis corresponding to its fundamental frequency, and the log-scale vertical axis indicates the cosine similarity between C-major and the tone specified on the horizontal axis. Note that the peaks corresponding with high-affinity tones have non-zero width, which accounts for why different tuning systems, including the modern equal-temperament system, are tolerable to listeners. The two profiles shown here are unnormalized and displayed on a log scale to highlight the effect of the choice of the attenuation and peak width on the resulting profiles. **(a)** Our model parameterized by *A*(*ϕ*,n) = *n*^–1^, *γ*_0_ = 0.01, and *N* = 50. **(b)** Our best fit model to the data of Krumhansl and Kessler (1982).

Figure 4 shows the key affinity of a tone with C-major, sweeping that tone continuously from C to C’. In the figure, the equal-tempered chromatic scale is shown on the horizontal axis. Peaks corresponding with the tones of the tonic triad can be clearly seen, and it is to be noted that these and the other peaks corresponding with chromatic scale tones do not always exactly align with the corresponding grid line. This is because they are fundamentally based on just intonation, given by harmonic multiples. However, because the peaks in the plot have width greater than zero, there is room for variability, which means that different tuning systems, including equal temperament, can be correctly captured by this one model.

We can compare our model with empirical data gathered and analysed by Krumhansl and Kessler (1982). In their experiment, Krumhansl and Kessler first played a major or minor chord followed by a single tone. They then asked participants to rate how well the tone fit musically with the previously played chord. They found strong correlation across all tonics of major and minor chords in the data, and so were able to define major and minor key affinity profiles across the 12 semitones.

**Figure 5:**
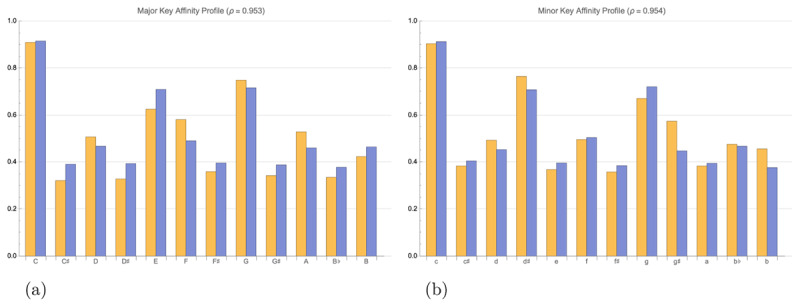
Comparison of the empirically measured key profiles of Krumhansl and Kessler (1982) (in orange) with the key affinity profile of our model (in blue). Across both profiles the correlation *ρ* = 0.950. **(a)** Key affinity profile of C-major. *ρ* = 0.953. **(b)** Key affinity profile of C-minor. *ρ* = 0.954.

Figures 5a and 5b compare the key affinity predicted by our best fit model (*γ*_0_ = 0.0309, |*d*_2_| = 0.672, and |*d*_3_| = 0.420 when *ϕ*_1_ = 1 in Equation 33) with the profiles measured empirically by Krumhansl and Kessler (1982), attaining high correlations of 0.953 for the major key profile, 0.954 for the minor key profile, and 0.950 across both profiles. These correlation values are competitive with other models of key affinity and shown in Table 1a, as originally compiled by Milne et al. (2015). The variable weighting of |*d*_2_|, and |*d*_3_| is most similar to model C of Milne et al. (2015). Constraining our model in the same way as Milne et al.’s models A, B, and C resulted in very similar, but slightly lower correlations, so we only present our model with separate |*d*_2_| and |*d*_3_|.

**Table 1:** Comparison of key affinity and inter-chord distance between our model and models reported elsewhere. **(a)** Correlation of selected models of key affinity with the major and minor profiles of Krumhansl and Kessler (1982), reproduced from Milne et al. (2015). **(b)** Correlation of selected models of inter-chord distance with empirical profiles of perceived triadic distance, reproduced from Milne and Holland (2016).


(a)			

KEY AFFINITY	BOTH	MAJOR	MINOR

Milne15c	.96	.98	.97

Lerdahl88	.95	.98	.95

Parncutt89	.95	.99	.94

**This paper**	**.95**	**.95**	**.95**

Parncutt11a	.93	.94	.95

Milne15b	.92	.98	.97

Milne15a	.91	.96	.93

**(b)**			

**INTER-CHORD DISTANCE**		**CORRELATION**	

Tonnetz		.92	

Spectral Pitch Class		.91	

**This paper**		**.90**	

Transformational		.83	

Minimal Voice Leading		.72	

Standard Voice Leading		.62	

Hamming		.88	


### 4.3 Inter-Chord or Inter-Key Distance

In Section 4.2, when measuring key affinity, a comparison was made between each pitch and a given key. Now, if two keys have similar affinity profiles, it stands to reason that they should be perceived as similar themselves. Krumhansl and Kessler (1982) use this idea to generate their key distance space. Therefore, in order to measure the similarity between two keys, instead of comparing between a pitch and a key, we will *directly* compare two keys (triads) using the inner product between their harmonic spectra. That is, we associate a resonance spectrum *f* composed of resonances with frequencies corresponding to the major (or minor) triad of the key, exactly as before, and another resonance spectrum *g* composed of resonances with frequencies equal to the major (or minor) triad of the other key. For instance, to compare the C-major key to the C-minor key, we would associate one with the frequencies of C, E, and G and the other with C, E♭, and G, following the musicological approach.

**Figure 6:**
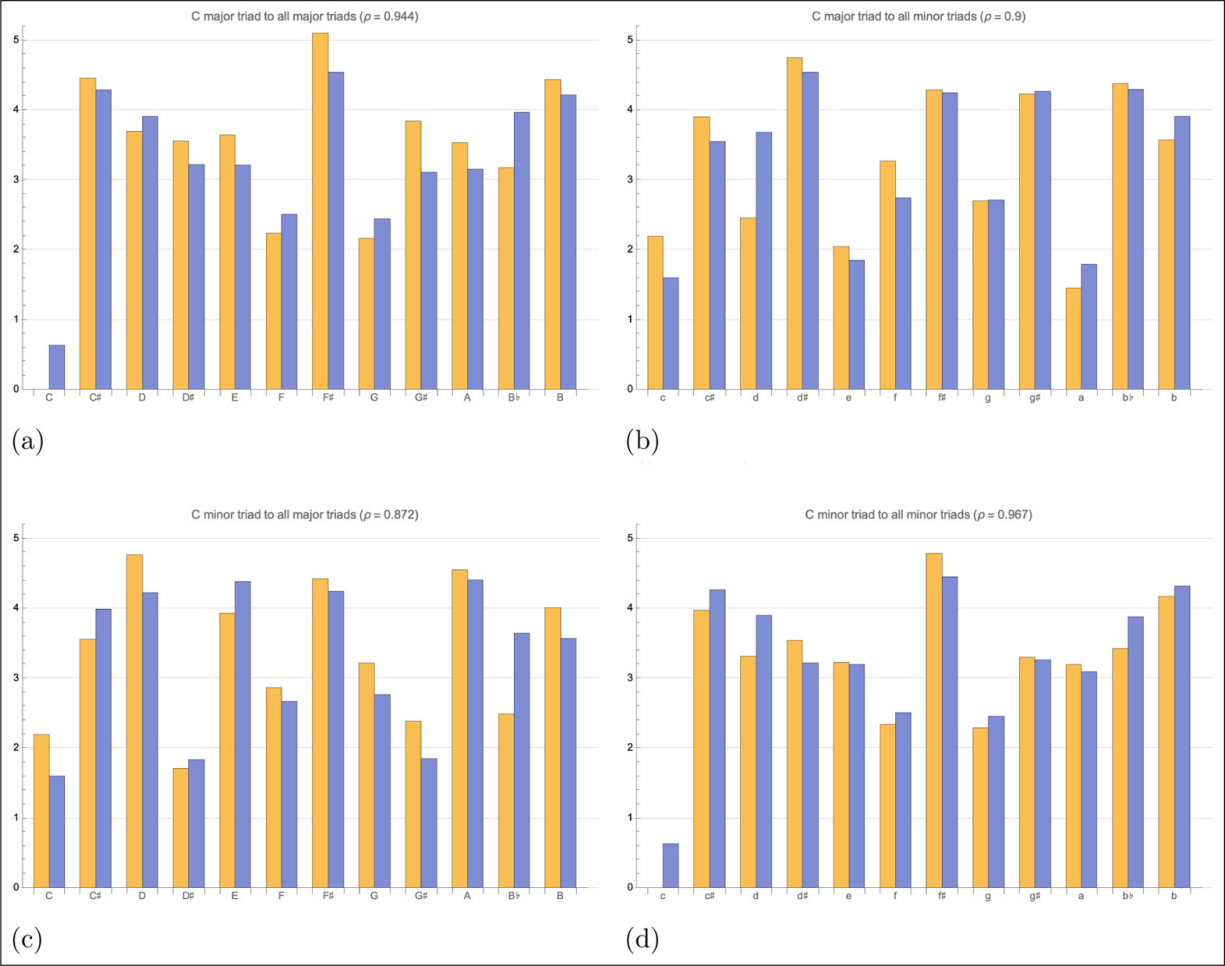
Comparison of inter-key distances derived from the 4-dimensional empirical model of Krumhansl and Kessler (1982) (in orange) with the inter-key distances of our model (in blue). Across all profiles correlation *ρ* = 0.916. The non-zero intercept seen in Figures 6a and 6d is an artifact of the normalization used to visualize the profiles, and so has no effect on the correlation. **(a)** Inter-key distance profile between C major and all major keys. *ρ* = 0.944. **(b)** Inter-key distance profile between C major and all minor keys. *ρ* = 0.900. **(c)** Inter-key distance profile between C minor and all major keys. *ρ* = 0.872. **(d)** Inter-key distance profile between C minor and all minor keys. *ρ* = 0.967.

Again, we fit the model to the empirical data of Krumhansl and Kessler (1982), though with the slight difference of converting the cosine similarity to a cosine distance of Equation 13. Similarly to the key affinity profiles, the inter-key distance profiles of our best fit model (*γ*_0_ = 0.0402, |*d*_2_| = 0.925, and |*d*_3_| = 1 when *ϕ*_1_ = 1 in Equation 33) are highly correlated with the empirically-derived 4D toroidal model of Krumhansl and Kessler (1982), as shown in Figure 6. The high correlation, *ρ* = 0.916 across all pairs of major and minor triads, suggests that our resonance space effectively models the four-dimensional geometry of the Krumhansl-Kessler empirical data, suggesting in turn that the resonance space and the harmonic operator constitute a valid underlying model of this aspect of music perception, and that it forms a candidate hypothesis (at some level of abstraction) for brain function during perception.

Due to the similarity in approach with Milne and Holland (2016), we compared our inter-chord distance model with their data of empirically measured perceived triadic distances (*γ*_0_ = 0.0402, |*d*_2_| = 0.925, |*d*_3_| = 1, with *ϕ*_1_ = 1 and *N* = 12), resulting in a correlation of *ρ* = 0.897. This correlation is compared with others in Table 1b Milne and Holland (2016).

## 5 Discussion

### 5.1 Overview

In this article, we have demonstrated how relationships between resonance spectra and harmonic spectra models how humans perceive harmony. As Milne has shown, this kind of approach can also provide explanatory models of key affinity, inter-chord (inter-key) distance, and pitch perception, in ways that are in principle naturally implementable in neural circuits. Furthermore, since all of these different types of perception occur concurrently when listening to music, it is reassuring that they can all be described using essentially the same mechanism. Moment by moment, *the same resonance spectrum* may be viewed in terms of the key affinity, inter-key/inter-chord distance, the fundamental frequencies of the pitches, and more. Though all of these are qualitatively different, they can be described using the same formalism. In terms of the model itself, everything is expressed in terms of just two key operations: the inner product of the resonance space and the harmonic operator *H*.

It is important to understand that this is an idealized model. The mathematics is based on ideal harmonic spectra, and the real world is not so uniform. Further work is required before the model can be used directly on resonances taken from audio waveforms; however, in principle, the harmonic operator works the same on the complicated spectra of natural, even inharmonic sounds, as it does on the simple, fundamentals-only spectra used in this article. What is relevant here is that the *geometry* of the resonance space effectively models pitch perception, which will remain the case even in the face of noisy data.

### 5.2 Future Work

In past work, we have studied the information dynamics of sequence in music (Pearce and Wiggins, 2012; Hedges and Wiggins, 2016; Hedges, 2017), mostly using the IDyOM cognitive model (Pearce, 2005). This work was based on predefined musical features that defined the representation space, though the model was capable of doing some predefined operations to choose combinations of features from the existing ones. Pearce (2005) and Hedges and Wiggins (2016) showed how the statistical properties of these features could be used to choose between them in terms of their utility in the cognitive model: how effectively did they assist encoding the necessary information? We call this the principle of information efficiency (Wiggins, 2020).

By contrast, in IDyOT we are working within a representation space that is highly general, without preconceptions of what features should be, musical or otherwise. In this article, the harmonic operator demonstrated how an operator can be designed in order to study some well-specified phenomenon. This has its merits, and other operators can be designed similarly to study other phenomenon. However, resonance space operators can also be discovered through empirical study or even learned directly from data.

Our next steps will involve building sequential statistical models from sonic data of both music and speech over the representations described in resonance space. This will allow us to derive the kind of feature representations that were hard-wired into IDyOM’s sequential models, thereby admitting the capacity to automatically segment sequences (cf. Pearce et al., 2010). We expect we will be able to simulate the perception of extended, dynamic time-based percepts. Wiggins and Sanjekdar (2019) have shown that it is possible to build larger structures out of smaller ones thus discovered in a way that corresponds with human perception. Similarly, we will extend the timebase of our system to much lower frequency ranges to bring in the models of rhythm and metre proposed by Forth et al. (2016), which also naturally lends itself to the resonance space representation.

With this sequential approach, we may think of moments of perception as points in resonance space. Thus, a sequence of such moments is a temporal trajectory through the space. That trajectory is itself a waveform, and can therefore also be represented in a resonance space, and so on upwards. Our strong hypothesis would suggest that such a construction is, in itself, a construction of meaning. So, the geometric structure of resonance space now becomes the primary means of categorizing and abstracting in IDyOT: categories are represented by regions, and abstractions are functionals of temporal trajectories.

## 6 Conclusion

In this paper, we have presented a cognitive model of music perception based on a mathematical structure called resonance space, in which fragments of sound are represented as linear combinations of atomic oscillatory functions called resonances. We have formalized resonance space as a Hilbert space whose inner product is used as the means to measure perceptual similarity between the spectra. We have shown how musical percepts and concepts such as tones, chords, and keys can be represented as distinguished elements of resonance space and constructed via the harmonic operator which maps resonances to a corresponding series of overtones. We have shown how this representation, combined with the inner product-based similarity, can be used to model perceptual phenomena including key affinity and chord and key distances, and have validated our results against corresponding empirical and musicological data. This harmonic spectral approach to the representation and comparison of musical percepts and concepts has its origins in the work of Jean Phillippe Rameau, and is distinguished from existing models by the choice of resonance as its spectral primitive and its general mathematical formulation.

This work is part of a broader research program which seeks to understand how psychological phenomena arise from, and are related to, physiological phenomena. The domain of music, in which the connection between physical stimulus and neurological response is now established, is used as a point of departure. We have given a speculative account of the relationship between our mathematical model of music perception and a possible neurophysiological implementation, starting from the observation that resonance spectra are consequence of modelling neural dynamics as a dynamical system, proceeding to suggest how the structure and operations of resonance space may correspond with the tonotopy, topology, and function of neuronal assemblies. The results of this research provide evidence that resonances indeed constitute a cognitively plausible proposal for the primitives of spectral knowledge representation, and the potential of resonance space to provide an appropriate mathematical foundation for sequential statistical models of sonic stimuli, and more broadly, the Information Dynamics of Thinking.

## Data Accessibility Statement

The publicly-available data used in this work comes from data previously published in the articles cited in the appropriate sections. Please consult those articles for access to the relevant data.

## Footnotes

The term *representation* here is potentially ambiguous. In psychology, *representation* usually means a mental record of one or more particular events. In AI, *representation* can mean this, but, more usually, it refers to a system or language by which knowledge is represented. For example, in AI, predicate logic is often used as a knowledge representation: a *language* used to represent knowledge. One can write down sentences in logic, and then these sentences become what a psychologist would usually mean by “a representation”. In this paper, we use the AI terminology.

The exact meaning of “similar” here will be the subject of future research.

There is a variety of functional forms of the Fourier transform that are used by convention in different disciplines; they are all equivalent as long as consistency is maintained. We elect to use the convention popular in the physics literature: \[{\cal F}[x(t)] = f(\omega): = {\textstyle{1 \over {\sqrt {2\pi } }}}\int_{ - \infty }^\infty x (t){e^{i\omega t}}dt\].

The signal can be considered as causal for *t* ≥ 0 and anti-causal for *t* ≤ *T*, i.e. causal-in-reverse from *t* = *T*. This allows the signal to be decomposed into resonances with both positive and negative decay, having the form of Lorentzian peaks in the frequency domain due to causality. Note that the original domain of *0* ≤ *t* ≤ *T* needs to be enforced when going from the frequency domain back to the time domain, since it diverges outside that interval.

In truth, the cosine distance as defined here is not a distance at all since it violates the triangle inequality. Even so, it behaves similarly to a distance measure, for our purposes.

The Fourier basis and Fourier transform are related, but categorically different things. The Fourier transform is a unitary operator that takes a function from the time domain to the frequency domain. The Fourier basis is an orthonormal sequence of oscillators that rotate an integer number of times over the interval [-π, π].

The term *frequency bin* is perhaps a misnomer, since it conjures an image of collecting nearby power from the frequency domain into its bin. In fact, it is solely the projection of the signal onto that precise frequency, not a range at all.

By tone, in this article we mean the fundamental frequency of a harmonic complex tone.

By “proxy”, here, we mean that we do not make strong scientific claims about this point, but rather treat the resonance system as a first approximation to what might later be a better model. Without approximations such as this, it is very difficult to find a starting point in complex modelling problems.
